# Supplementary material for: Serum biomarkers and anti-flavivirus antibodies at presentation as indicators of severe dengue
Source: PLoS Negl Trop Dis. 2023 Feb 27;17(2):e0010750. doi: 10.1371/journal.pntd.0010750 (PMC9997924; doi:10.1371/journal.pntd.0010750)
Supplement: S1 Fig — (PDF) [file pntd.0010750.s001.pdf]

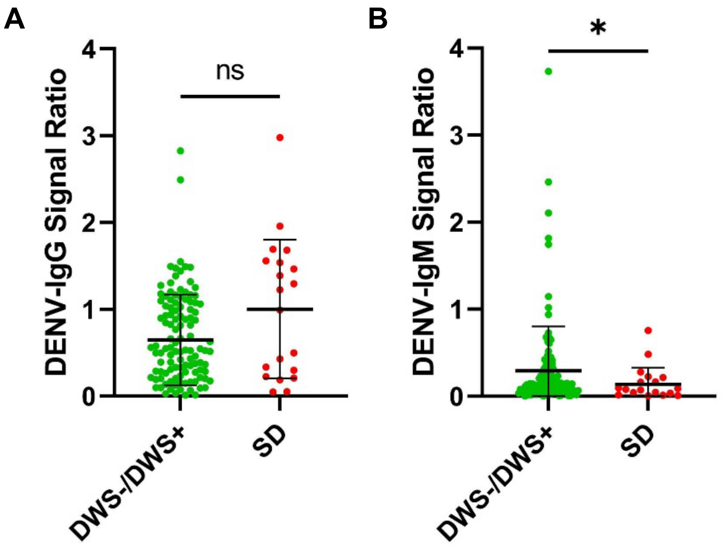

Figure S1. Distributions of DENV pGOLD IgG (A) and IgM (B) results by disease severity with bars representing mean and standard deviation. P-values are for overall ANOVA tests. ns, $P > 0.05$; *, $P \leq 0.05$.
