# Supplementary material for: Serum biomarkers and anti-flavivirus antibodies at presentation as indicators of severe dengue
Source: PLoS Negl Trop Dis. 2023 Feb 27;17(2):e0010750. doi: 10.1371/journal.pntd.0010750 (PMC9997924; doi:10.1371/journal.pntd.0010750)
Supplement: S1 Table — (PDF) [file pntd.0010750.s003.pdf]

Table S1. Characteristics of participants by severity category.

Characteristic	DWS-	DWS+	SD	p-value
Patients	55	67	23	
Gender, female (%)	38 (69.1)	43 (64.2)	6 (26.1)	0.001
Age, years, mean (st. dev.)	34 (17)	34 (19)	61 (19)	<0.001
Clinical site				
HVE	4	7	4	
IICS	1	5	-	
HC-IPS	50	55	19	
Presence of comorbidities	15 (27%)	19 (29%)	16 (84%)	<0.001
Days of symptoms, mean (st. dev.)	3.7 (1.7)	4.1 (2.0)	4.8 (1.7)	0.06
DENV rRT-PCR, positive	50 (91%)	60 (90%)	20 (91%)	0.96
Serotype				0.32
DENV-1	5 (10%)	9 (15%)	4 (20%)	
DENV-2	2 (4%)	7 (12%)	0 (0%)	
DENV-4	43 (86%)	43 (72%)	16 (80%)	
Negative	0 (0%)	1 (2%)	0 (0%)	
NS1, positive	31 (56%)	46 (69%)	21 (91%)	0.011
Warning signs				
Bleeding	-	34 (49%)	4 (17%)	
Fluid accumulation	-	7/67	6/22	
Shortness of breath	-	37/67	12/22	
Abdominal pain	-	30/67	4/22	

Missing data: Presence of comorbidities, DWS+ (1), SD (4); Warning signs, SD (1).

Abbreviations: HVE, Hospital de Villa Elisa; IICS, Instituto de Investigaciones en Ciencias de la Salud; HC-IPS, Hospital Central - Instituto de Previsión Social; st. dev., standard deviation.
