# Supplementary material for: Serum biomarkers and anti-flavivirus antibodies at presentation as indicators of severe dengue
Source: PLoS Negl Trop Dis. 2023 Feb 27;17(2):e0010750. doi: 10.1371/journal.pntd.0010750 (PMC9997924; doi:10.1371/journal.pntd.0010750)
Supplement: S2 Table — (PDF) [file pntd.0010750.s004.pdf]

Table S2. Serologic test results stratified by disease severity.

Serological Test	DWS-	DWS+	SD	p-value
pGOLD				
DENV IgM	27/54 (50.0%)	44/65 (67.7%)	7/20 (35.0%)	0.019
DENV IgG	47/54 (87.0%)	55/65 (84.6%)	18/20 (90.0%)	0.81
ZIKV IgM	1/54 (1.9%)	3/65 (4.6%)	0/20 (0%)	0.80
ZIKA IgG	6/54 (11.1%)	13/65 (20.0%)	4/20 (20.0%)	0.24
ELISA				
DENV IgM	14/55 (25.5%)	26/67 (38.8%)	5/23 (21.7%)	0.18
DENV IgG	49/55 (89.1%)	57/67 (85.1%)	22/23 (95.7%)	0.20
