# Supplementary material for: Serum biomarkers and anti-flavivirus antibodies at presentation as indicators of severe dengue
Source: PLoS Negl Trop Dis. 2023 Feb 27;17(2):e0010750. doi: 10.1371/journal.pntd.0010750 (PMC9997924; doi:10.1371/journal.pntd.0010750)
Supplement: S3 Table — (PDF) [file pntd.0010750.s005.pdf]

Table S3. Comparison of pGOLD and Vircell ELISA detection of anti-DENV IgM and IgG in acute-phase samples.

A

IgM ELISA	pGOLD (IgM)			
	**Positive**	**Negative**	**Total**
**Positive**	39	4	43
**Negative**	38	55	93
**Total**	77	59	136

B

IgG ELISA	pGOLD (IgG)		
	**Positive**	**Negative**	**Total**
**Positive**	112	11	123
**Negative**	4	4	8
**Total**	116	15	131
