# Supplementary material for: Serum biomarkers and anti-flavivirus antibodies at presentation as indicators of severe dengue
Source: PLoS Negl Trop Dis. 2023 Feb 27;17(2):e0010750. doi: 10.1371/journal.pntd.0010750 (PMC9997924; doi:10.1371/journal.pntd.0010750)
Supplement: S4 Table — (PDF) [file pntd.0010750.s006.pdf]

Table S4. Laboratory values by disease severity.

Laboratory Test[a]	DWS-/DWS+	SD	p-value
Bilirubin, mg/dL	0.49±0.26 (64)	2.51±2.11 (11)	0.010
Blood Creatinine, mg/dL	0.85±0.22 (61)	3.76±2.29 (13)	<0.001
Chymase, ng/mL	1.2±5.8 (122)	30.0±28.9 (23)	<0.001
Hematocrit, %	39.4±4.5 (117)	35.9±8.2 (23)	0.066
Hemoglobin, g/dL	13.4±1.5 (117)	12.0±3.1 (23)	0.046
Leukocytes, /µL	4,692±2,155 (117)	12,192±10,765 (23)	0.004
LBP, ng/mL	11,917±5,030 (85)	18,766±5,510 (23)	<0.001
Lymphocytes, /µL	1,370±1,196 (117)	1,430±1,798 (23)	0.88
Lymphocytes, %	30.6±17.9 (117)	12.6±10.1 (23)	<0.001
Neutrophils, /µL	3,079±2,320 (117)	10,375±9,186 (23)	0.001
Neutrophils, %	62.5±21.7 (117)	83.1±8.8 (23)	<0.001
Platelets, /µL	152,462±72,110 (117)	68,364±60,249 (23)	<0.001
SGOT, IU/L	58.0±45.2 (74)	102.8±66.6 (10)	0.007
Viral Load, log_{10} c/mL serum	6.13±1.7 (122)	6.55±1.49 (23)	0.34

Abbreviations: c, copies; IU, international units; LBP, lipopolysaccharide binding protein; SGOT, serum glutamic oxaloacetic transaminase

[a] Presented as mean ± standard deviation (n, number of participants with data)
