# Supplementary material for: Serum biomarkers and anti-flavivirus antibodies at presentation as indicators of severe dengue
Source: PLoS Negl Trop Dis. 2023 Feb 27;17(2):e0010750. doi: 10.1371/journal.pntd.0010750 (PMC9997924; doi:10.1371/journal.pntd.0010750)
Supplement: S5 Table — (PDF) [file pntd.0010750.s007.pdf]

Table S5. Laboratory values by disease severity categories.

Laboratory Test[a]	DWS-	DWS+	SD	p-value
Bilirubin, mg/dL	0.46±0.19 (23)	0.51±0.29 (41)	2.51±2.11 (11)	0.013
Blood Creatinine, mg/dL	0.91±0.17 (25)	0.81±0.25 (36)	3.76±2.29 (13)	<0.001
Chymase, ng/mL	1.4±8.1 (55)	1.0±2.9 (67)	30.0±28.9 (23)	<0.001
Hematocrit, %	39.4±4.2 (52)	39.3±4.8 (65)	35.9±8.2 (23)	0.17
Hemoglobin, g/dL	13.4±1.5 (52)	13.4±1.6 (65)	12.0±3.1 (23)	0.12
Leukocytes, /µL	4,848±2,167 (52)	4,568±2,155 (65)	12,192±10,765 (23)	0.007
LBP, ng/mL	11,116±4,561 (44)	12,777±5,413 (41)	18,766±5,510 (23)	<0.001
Lymphocytes, /µL	1,340±926 (52)	1,396±1,387 (65)	1,430±1,798 (23)	0.96
Lymphocytes, %	30.0±16.1 (52)	31.2±19.3 (65)	12.6±10.1 (23)	<0.001
Neutrophils, /µL	3,102±1,840 (52)	3,060±2,656 (65)	10,375±9,186 (23)	0.003
Neutrophils, %	61.7±15.7 (52)	63.1±25.6 (65)	83.1±8.8 (23)	<0.001
Platelets, /µL	170,500±63,606 (52)	138,031±76,325 (65)	68,364±60,249 (23)	<0.001
SGOT, UI/L	37.5±27.9 (28)	70.5±49.3 (46)	102.8±66.6 (10)	<0.001
Viral Load, log_{10} c/mL serum	6.21±1.50 (52)	6.06±1.88 (65)	6.55±1.49 (23)	0.57

Abbreviations: c, copies; IU, international units; LBP, lipopolysaccharide binding protein; SGOT, serum glutamic-oxaloacetic transaminase

[a] Presented as mean ± standard deviation (n, number of participants with data)
