# Supplementary material for: Serum biomarkers and anti-flavivirus antibodies at presentation as indicators of severe dengue
Source: PLoS Negl Trop Dis. 2023 Feb 27;17(2):e0010750. doi: 10.1371/journal.pntd.0010750 (PMC9997924; doi:10.1371/journal.pntd.0010750)
Supplement: Attachment — Submitted filename: Response to reviewers v1.0.docx [file pntd.0010750.s008.docx]

**Reviewer Comments**

**Reviewer #1**

**Rather than using the brand name of the methods in the abstract "pGold assay", please write the type of assay used so it is clear what information was gained without reading the methods section. (e.g. DENV and ZIKV IgG/IgM?) The brand name is more appropriate for the methods section.**

The abstract has been revised to read (lines 40-43),

“Testing for anti-dengue virus IgM and IgG and serum biomarkers (lipopolysaccharide binding protein and chymase) was performed on acute-phase sera in plate-based ELISAs; in addition, a multiplex ELISA platform was used to measure anti-dengue virus and anti-Zika virus IgM and IgG.”

**The abstract should clarify whether the selected cases for biomarkers were confirmed dengue. As written currently, it suggests that all "suspected arboviral infections" were used to model dengue.**

In the revision (line 36), it is now stated that 145 confirmed dengue cases were selected for the current study.

**The aspect of defining dengue based on "strong epidemiological link" is unclear and maybe a weak definition depending on the reasoning. At least this is non-standard. More information is needed here to fully consider this inclusion criteria.**

We appreciate this concern by Reviewer #1. The “Case Definition” section in the Methods has been clarified, and it is not emphasized that this criterion applied only to a single participant in the dengue without warning signs category. This section of the Methods now reads as follows (lines 196-199):

“Dengue cases were defined as individuals who met inclusion criteria for the parent study and had 1) detectable DENV RNA in the ZCD and/or DENV multiplex rRT-PCR or 2) detection of DENV NS1 by rapid test. For a single participant with DWS-, dengue was defined based on clinical presentation and a strong epidemiologic during a large was of DENV-4 cases.”

Additionally, this point is emphasized in the Results (lines 245-246).

**It is also unclear in the methods if the collection of "acute" samples preceded the final diagnosis involving severity? Even if not, it should be stated clearly.**

Samples were taken at the initial visit where participants were given a diagnosis of severity and acute-phase samples were taken. Some cases evolved to a more severe category over the course of their illness, and final classifications were upgraded to the most severe category that could be confirmed based on medical records. A statement clarifying this has been added to the Methods (lines 151-152).

**Is it not odd that most of the cases are DENV IgM-negative when this should be induced during acute dengue, including acute secondary dengue?**

In acute secondary dengue, anti-DENV IgM antibodies are relatively low and do not start to increase until later in the course of illness, after around day 5. As we limited the study population to those with 7 or less days of symptoms, time points at which anti-DENV IgM may have been higher were not include. Our results are consistent with previous findings of poor anti-DENV IgM sensitivity in acute dengue cases in Paraguay (predominantly DENV-1, ref 52).

**The discussion does state that this was "not a prospective study", but it would be important to clearly define it as a retrospective study in the methods, if appropriate.**

This was a cross sectional study to provide exploratory analysis of these biomarkers in our population. This has been clarified in the methods section (lines 147-149).

**Line 111- are these anti-DENV and anti-ZIKV binding titers or are they neutralizing titers? Because DENV antibody cross-reactivity can lead to positive signals for ZIKV even in populations that have not been exposed to the virus. Some clarification would be helpful here.**

These are binding titers, but these have been shown to correlate with DENV neutralizing titers. We agree with this comment about cross-reactivity, and indeed, in our patient population, anti-ZIKV antibodies appear to predominantly result from cross-reactivity in the setting of high anti-DENV titers. This is discussed in greater detail in Ref 59

**The assessment of anti-NS1 IgM levels is very interesting but did the authors measure NS1 levels itself?**

NS1 levels were not measured for this study as NS1 antigen was detected using a rapid, lateral flow assay.

**For those patients who are ZIKV NS1-IgM positive, do they have neutralizing antibodies against ZIKV? i.e. do they just have more cross-reactive DENV antibodies or are they truly ZIKV-immune?**

These are likely cross-reactive anti-DENV antibodies in our participants. This has been clarified in the Results (lines 322-325). This section now reads,

“As anti-DENV antibodies detected in the pGOLD assay target whole viral antigen, interactions between chymase and antibodies directed against the NS1 protein of ZIKV were investigated for their association with SD. These antibodies are detected in the pGOLD assay and predominantly represent cross-reactive anti-DENV antibodies in this population [59].

**Could the authors please add the data controlling for day post-symptom onset (data not shown)? It seems clear that time should be a co-variate.**

We agree with Reviewer #1 that day post-symptom onset would be expected to impact biomarker results. We evaluated this as an effect modifier in both the serologic and laboratory domain models, but this did not hold significance and was removed from the final models. This likely occurred as day post-symptom onset was controlled for, in part, by study design limiting sample selection to individuals with ≤7 days of symptoms at presentation. This is not a priori associated with dengue severity so is not a confounder, and the use of a single sample per individual may have also impacted power to see changes over the first few days post symptom onset. This has been clarified in the Results (lines 269-271).

**Figure 1 Panel e- The scale of the y-axis appears wrong. Please check all units/scales**

Axes were checked, and errors in the y-axis were corrected in the article and supplement.

**Line 86: "Chymase release from mast cells occurs in the presence of DENV and may be increased by pre-existing anti-DENV IgG antibodies or blocked by antibodies against viral non-structural protein 1 (NS1) [33, 42]. The first part of this sentence has been shown in humans and mice, whereas the second part relies on a single citation from an immune compromised mouse study. This should be clarified since the second aspect in mice has not been reproduced independently or shown in humans or with human cells.**

This has been clarified in the revision. This section of the Introduction (lines 96-99) now reads,

“Chymase release from mast cells occurs in the presence of DENV and may be increased by pre-existing anti-DENV IgG antibodies (36, 37). According to a single study in mice, chymase release may be blocked by antibodies against viral non-structural protein 1 (NS1) (45).”

**Line 289, this is consistent with citation 34 as well.**

We believe this was in reference to line 89 in the original submission, and this citation has been updated in the revision (line 95).

**Line 343- Is model system indicated correct? Is it not in mice?**

It was a dengue mouse model and an evaluation of mast cell degranulation. This has been corrected in the revision (line 375).

**This is a very controversial statement (Line 380) "However, as many pathways associated with clinically severe dengue appear linked to NS1, including chymase release" Actually only one study suggests an association between NS1 and chymase release, whereas most studies examined the role of the virus itself or antibody-mediated triggering of mast cells by immune complexes. In fact, in many studies NS1 levels do not correlate with disease severity even when chymase levels do. It is plausible that NS1-antibodies could contribute to mast cell activation during dengue but it's not well supported and even incorrect to suggest this is the only mechanism.**

These are very valid concerns noted about this line in the text, and indeed, we never intended to suggest that elevated NS1 provided a unifying mechanism for the development of severe dengue. This sentence has largely been removed from the revision, and it has been replaced with a general statement about the potential benefits to an improved antigen diagnostic in combination with a prognostic test. This line (412-414) of the Discussion now reads,

“An improved antigen diagnostic in combination with a prognostic test may then increase DENV detection, identify individuals at high risk for SD, and facilitate future trials for clinically severe dengue.”

**There are many occasions where the writing is a bit confusing and there could be errors in the text that need to be checked carefully, but overall, the data appears to be well presented and clear.**

We appreciate this concern and believe that the significantly revised version of this article has clarified key elements of the text.

**This is a valuable study that reproduces some of the key biomarkers of severity that have been identified in prior literature, independently, in a new dengue cohort. It is equally important to share the results where the authors' data is negative, showing a statistical difference between groups but poor predictive value in their model. However, in the absence of mechanistic data the authors have relied on interpretations from the literature for discussion, which sometimes are not well supported. This study could be brought to publishable form by improving the writing and description of the methods, adding some data that was not shown (e.g. NS1 concentrations, time based analyses), and toning down over-interpretations relating to mechanisms of disease that are assumed based on other literature but not explicitly proven here.**

We appreciate the constructive comments from Reviewer #1. We have addressed concerns noted in each of the sections of the revision and toned-down interpretations found in the Discussion as detailed above.

**Reviewer #2**

**Overall, this is an interesting and clinically relevant exploratory study aiming to identify biomarkers of dengue severity at a single time-point. It is valuable to read about severe dengue and DENV4 cases in Latin America, both of which are relatively rare in the literature. The manuscript is well-written and easy to follow. I thought the conclusions were clear and fairly written. I appreciated that they highlighted potential reasons for not observing differences in viral load between groups, and the discussions of the limitations were thorough and helpful.**

**- The objective is to evaluate the biomarkers of dengue severity at a single timepoint. The study is exploratory and no specific hypotheses were stated.**

**- This cross-sectional observational cohort is appropriate for an exploratory study.**

**- The population was generally clearly described, but I do have a few follow-up questions on the population.**

We appreciate these comments from Reviewer #2.

**In line 154, authors mention that samples were tested for ZIKV, chikungunya and DENV, but they do not state if any subjects tested positive for ZIKV and/or CHIKV. Can they clarify whether any participants had coinfections and if so, how coinfections were managed?**

A statement was added in Results to clarify that no Zika or chikungunya virus infections were detected, nor were coinfections by two DENV serotypes (lines 250-251).

**In line 142, they state that all SD cases were included, and a mixture of DWS- and DWS+ cases was then selected to achieve a representative distribution of participants. Can they state how many individuals were in the full cohort? And, how did they select participants for the subcohort? Was a statistical package used for this? Since this was exploratory, they did not include power calculations. However, can they state why they chose n = 55 participants with DWS- and n = 67 participants with DWS+? Was there some significance to these numbers?**

The full cohort includes 1566 individuals with suspected arboviral illness cases, and this information has been added in Methods (line 148). Additional information has been added to clarify that cases selected for the subcohort were limited based on sample volume and availability of clinical and demographic data (lines 158-159). As noted by the reviewer, this was an exploratory analysis to determine the potential utility of these biomarkers, which had not been studied in our patient population or, to our knowledge, among dengue cases in the Americas.

**One other question I had about the methods was can they explain how they created the comorbidity variable included in the models? It looks like in the model described in table 2, the comorbidity variable was dichotomous: present/ absent. Was comorbidities also coded as a dichotomous variable in the chymase interaction model (lines 287 - 296)?**

Comorbidities were initially defined as present or absent, but for all logistic regression models in the revision, this has been changed to a discrete variable. This is now shown in Table 1, and the new results of the logistic regression are shown in Table 2. For t-test calculation in the chymase analysis, comorbidities remained coded as a dichotomous variable.

**From the models, it looks like the effect size of comorbidities is quite large, and I think it would be valuable if the authors could delve into this variable a bit more. Specifically, from the table 1 footnote, it looks like 32 had hypertension, 14 had diabetes and 30 had other. What were the other comorbidities? It looks like the models may have just considered presence/absent of comorbidities, but were there differences in the number of comorbidities between people with SD vs DWS-/DWS+? Was a specific comorbidity more predictive? For example, did all participants with SD have diabetes but an equal number of participants in both groups have hypertension? From a clinical perspective, it is nice to know what comorbidities to look out for, and if your patient does have a certain comorbidity, how might that relate to the presence of SD?**

We appreciate this recommendation from Reviewer #2. For the revision, comorbidity was converted to a discrete variable as a count of how many comorbidities each patient had, and we have provided more detail on the comorbidities in Table 1. Logistic regression analysis was performed with comorbidity as a discrete variable, which generated an OR of 2.09 with a narrower 95% CI of 1.26-3.48. Our data do not demonstrate a disproportionate risk from a particular comorbidity. Rather, the logistic regression analysis indicates that risk increases with each additional comorbidity, likely as a marker of overall patient health when they develop dengue. For all t-tests, such as the chymase analysis, comorbidity was left as a binary categorical variable.

**Line 248: can authors clarify does the pGOLD assay correlates with DENV1-4 neutralizing titers (i.e. geometric mean titer)? Or does it correlate with the neutralizing titers against a DENV2 since it is based on antibodies against DENV2?**

Results from the pGOLD assay correlate with DENV1-4 neutralizing titer. It is not a specific correlation with neutralizing antibodies against DENV-2.

**Line 274: In their model presented in Table 5, I wasn't sure why the authors focused on lymphocyte percentage when there was no actual difference in lymphocyte count. In contrast, there was a difference in both neutrophil count and percentage between the two groups. So, in this cohort, aren't the differences in neutrophils really driving the observations, and the difference in lymphocyte percentage is just a response to difference in neutrophils? For example, if there are more neutrophils, then the neutrophil percentage goes up, which pushes the lymphocyte percentage down. Can the authors clarify why they focused on lymphocyte percentage rather than neutrophil count and percentage?**

While lymphocyte percentage and neutrophil percentage both had strong crude associations with disease severity and little missing patient data, we could not evaluate both in the same model as they were predictably highly collinear. This latter point is clarified in the Results (lines 293-295). We decided to focus on lymphocyte percentage as that was more classically associated with severe dengue in the literature. The neutrophilia observed in our population is interesting, but this is also commonly observed in bacterial processes. As a result, this was not felt to be a good marker for severe dengue.

**Additionally, can the authors explain why they picked lymphocyte percent, platelet count, and chymase for table 5? I understand that bilirubin, SGOT and creatinine had many missing values but what about LBP, neutrophils, hematocrit? I didn't quite understand why the authors went from a multivariate logistic regression with multiple variables (line 271) to a logistic regression with 3 variables (line 274-275). Did they see something in the multivariate regression that motivated the smaller model?**

Lymphocyte percent, platelet count, hematocrit, LBP, and chymase were evaluated for the final laboratory domain model. They had the most complete data for every patient. Lymphocyte percentage and neutrophil percentage could not both be included as they were highly collinear. The five variables listed above were evaluated in a model and those that had non-significant regression coefficients were dropped from the model. Statements clarifying this were added to the Results, and this section (lines 290-298) now reads,

“… crude associations with SD were calculated for all variables by binomial regression (Table 4), and variables evaluated in the laboratory domain multivariable logistic regression were limited to lymphocyte percent, platelet count, hematocrit, LBP, and chymase. These analytes displayed crude associations with SD, had sufficient data points to maintain model strength, and were not collinear with each other (e.g., hemoglobin and hematocrit, neutrophil and lymphocyte percent). After evaluating these five variables, those with non-significant regression coefficients were removed (LBP and hematocrit). In the final binary logistic regression model, lymphocyte percent, platelet count, and chymase were found to be associated with SD with a very good model fit (AUROC statistic, 0.95; Table 5).”

**Line 298 - 307: can the authors explain why they chose to look for interactions between ZIKA IgM and IgG and chymase? It seemed that Zika IgM was associated with chymase OR, but none of the patients with severe dengue had a positive Zika IgM. In contrast, they state that anti-DENV IgG levels were higher in those with severe dengue (line 349). Additionally, they state that Dengue IgG is associated with increased chymase in other studies (line 88-89 and line 342). Given these findings, wouldn't it make sense to also look for associations between anti-DENV IgG and chymase?**

This portion of the analysis was performed to evaluate the finding that anti-NS1 antibodies may decrease mast cell degranulation. That finding was observed in a mouse model and has not been replicated in human cases. We took advantage of the fact that the pGOLD multiplex assay includes NS1 from ZIKV to evaluate anti-NS1 antibodies to a related flavivirus in relation to chymase levels and SD. This has been clarified in the Results (lines 321-325),

“As anti-DENV antibodies detected in the pGOLD assay target whole viral antigen, interactions between chymase and antibodies directed against the NS1 protein of ZIKV were investigated for their association with SD. These antibodies are detected in the pGOLD assay and predominantly represent cross-reactive anti-DENV antibodies in this population [59].

**In Line 349, they state that higher levels of anti-DENV IgG and undetectable anti-DENV IgM were associated with SD in their population. However, Table 3 doesn't really show these relative differences, especially in IgG levels. Instead, it only shows whether the antibodies were present or not. Instead of a table, it might be more informative to have a figure with serology distributions for each group, as is shown for the laboratory values in figure 1.**

A new Figure S2 has been added to show the distribution of IgM and IgG results in the pGOLD by severity. Qualitative antibody results were maintained in the main paper as this provides for comparisons between detection methods.

**Table S4 and S5 have a superscript a that says "missing data," but that superscript does not seem to have any information.**

We apologize for this oversight. These tables have been updated to include the number of participants from whom data was available.

**Line 398: can they really say that these findings will aid in the early detection of potentially severe dengue cases? They did not state that individuals with severe dengue were particularly early in their course when they had these abnormalities. I think the most they can conclude is that these variables might aid in early detection and should be evaluated in prospective trials.**

We appreciate this comment, and the concluding remark (lines 427-429) has been toned down to read, “…our findings may aid in the early detection of potentially severe dengue cases…”.

**Reviewer #3**

**This study aims to identify markers for the early detection of potentially severe dengue cases and inform the development of new prognostics for use in acute-phase and serial samples. The results are not entirely new as decreasing platelet and increasing chymase and low lymphocytes have previously been reported to be predictive of severe dengue. Limitations are the small sample size. The strength is the inclusion of a wide age range in order to identify age as a risk factor. It is also unique in that the cohort is a predominantly adult population whilst most studies are generally done in pediatric cohorts. Overall, the manuscript is very well written, highlights its limitations and the figures and tables are well done.**

We appreciate these comments from Reviewer #3.

**Abstract: Most readers would not know where Asuncion is, please add country.**

The abstract has been updated accordingly.

**How were these 145 participants selected from the "parent" study? How many subjects are in the parent study, and what criteria did you use to select 145 out of the total number? Was it consecutive recruitment, or selection based on certain inclusion criteria that were different to the inclusion criteria in the parent study?**

A statement was added clarifying that there were 1566 individuals in the full parent study (lines 147-149). To be included in the current study, participants had to have a confirmed dengue case, whereas the parent study only required acute illness with a selection of symptoms without a diagnosis.

**Why was chymase selected and not also tryptase?**

Both proteases, chymase and tryptase, are released by mast cells and have been studied as predictors and mediators of dengue severity. However, the preponderance of published data focuses on chymase, and studies that evaluate both as predictors of severe dengue have found similar results or perhaps slightly improved prediction with chymase (Furuta, et al., PLoS NTD, 2012; Rathore, et al., JCI, 2019). Therefore, we selected chymase over tryptase for the current study.

**Nice tables! Tables: shouldn’t abbreviations such as LBP not be spelled out in a legend?**

Thank you for this comment. Abbreviations used in the tables have now been spelled out in the accompanying legends.

**What is the significance of your findings? How should they translate into clinical practice? We already know that chymase, platelets and lymphocytes are predictive, so what is the added value of your study? The conclusions should therefore be stronger: maybe a point of care test should be developed to test for chymase to help triage patients in the first days of illness?**

Previous data for chymase and LBP came from studies in Southeast Asia, and it has long been discussed that dengue severity may differ by region. It is therefore important to evaluate these markers in the Americas, and to our knowledge, this is the first such study. This is highlighted in the Discussion (lines 359-360 and lines 366-369). In addition, this article provides additional data on use of the pGOLD assay, which was evaluated as a potential means to translate critical work on preceding anti-DENV antibody titers into a prognostic that could be used at the acute presentation.

We agree that development of new diagnostic-prognostic devices could help with patient management and triage in the early phases of disease, and the closing statement in the Discussion has been strengthened to emphasize this point and respond to this comment. These lines (427-429) now read,

“Therefore, our findings may aid in the early detection of potentially severe dengue cases and serve as a basis for the development of new combined diagnostic-prognostics for use in acute-phase and serial samples from dengue cases.”

**Introduction: please refer to this recent review that provides an updated mapping of dengue globally over the past 30 years: Global burden for dengue and the evolving pattern in the past 30 years. Yang X, Quam MBM, Zhang T, Sang S. *J Travel Med*. 2021 Dec 29;28(8):taab146.**

We appreciate the recommendation of this reference, which also highlights higher rates of death and DALYs in patients 15-49 years old. This has been cited at multiple locations in the Introduction and specifically highlighted in the Discussion as Ref 5. This later portion (lines 341-344) now reads,

“Although clinically severe dengue often occurs among children [1, 3, 27], age among adults has been identified as a risk factor for poor outcomes, and in 2019, individuals 15-49 years of age accounted for more deaths and disability adjust life years lost than children [5, 20, 26, 49].”

**Also important to highlight that dengue is not only a problem of the endemic population but also increasingly of the travellers population: Dengue, chikungunya and Zika in GeoSentinel surveillance of international travellers: a literature review from 1995 to 2020. Osman S, Preet R. *J Travel Med*. 2020 Dec 23;27(8):taaa222.**

Dengue among returning travelers is mentioned in the Introduction to the revision, and this article has been cited. These lines (73-75) now read,

“Over the past 30 years, dengue incidence and associated deaths have increased both in those residing in and travelers returning from areas of endemicity [5, 6].”

**Maybe also highlight that contrary to common perception, also primary dengue can have fatal outcomes: Fatal outcomes of imported dengue fever in adult travelers from non-endemic areas are associated with primary infections. Huits R, Schwartz E. *J Travel Med*. 2021 Jul 7;28(5):taab020.**

This article is now cited in the Discussion (Ref 74). The edited lines (382-384) now read,

“This is consistent with findings in secondary dengue cases, though this is difficult to determine with certainty in acute-phase samples [3, 73], and SD can occur in primary dengue, particularly among adults experiencing a first infection [74].”

**Discussion: The authors should ideally also set their chymase results in relation to tryptase, and other biomarkers. A systematic review on biomarkers was published.**

1. **Markers of dengue severity: a systematic review of cytokines and chemokines. Lee YH, Leong WY, Wilder-Smith A. *J Gen Virol*. 2016 Dec;97(12):3103-3119.**
2. **Dengue virus-elicited tryptase induces endothelial permeability and shock. Rathore AP, Mantri CK, Aman SA, Syenina A, Ooi J, Jagaraj CJ, Goh CC, Tissera H, Wilder-Smith A, Ng LG, Gubler DJ, St John AL. *J Clin Invest*. 2019 Jul 2;129(10):4180-4193.**

These two references have been cited in new lines (369-372) of the Discussion section, which read,

“As markers of dengue severity, mast cell degranulation factors have demonstrated more consistent results that other potential markers such as chemokines and cytokines [34-36, 40, 70], tryptase may play a direct pathophysiologic role in endothelial permeability [68], and mast cells can be stabilized by available FDA-approved medications [35].”

The reference by Lee, et al., is now reference 70, and the reference by Rathore, et al., is now reference 68.

**Ref 1 is now quite old and could be updated by a similar more recent paper in the same journal with the same title:** **Dengue. *Lancet*. 2019 Jan 26;393(10169):350-363.**

This review, now reference 4 in the revision, has been cited at multiple locations in the Introduction and Discussion.
